# Item Features Interact With Item Category in Their Influence on Preferences

**DOI:** 10.3389/fpsyg.2020.00988

**Published:** 2020-07-23

**Authors:** Shiran Oren, Tal Sela, Dino J. Levy, Tom Schonberg

**Affiliations:** ^1^Sagol School of Neuroscience, Tel Aviv University, Tel Aviv, Israel; ^2^Department of Behavioral Sciences, School of Social Sciences and Humanities, Kinneret Academic College on the Sea of Galilee, Zemach, Israel; ^3^Coller School of Management, Tel Aviv University, Tel Aviv, Israel; ^4^Department of Neurobiology, The George S. Wise Faculty of Life Sciences, Tel Aviv University, Tel Aviv, Israel

**Keywords:** visual-features, preference, decision-making, fractal-art, snacks, faces, replications

## Abstract

Low-level visual features are known to play a role in value-based decision-making. However, most previous studies focused on the role of only a single low-level feature or only for one type of item. These studies also used only one method of measurement and provided a theory accounting for those specific findings. We aimed to utilize a different more robust approach. We tested the contribution of low-level visual features to value-based decision-making of three item types: fractal-art images, faces, and snack food items. We used two techniques to estimate values: subjective ratings and actual choices. We found that low-level visual features contribute to value-based decision-making even after controlling for higher level features relevant for each item category (for faces, features like eye distance and for food snacks, features like price and calories). Importantly, we show that, overall, while low-level visual features consistently contribute to value-based decision-making as was previously shown, different features distinctively contribute to preferences of specific item types, as was evident when we estimated values using both techniques. We claim that theories relying on the role of single features for individual item types do not capture the complexity of the contribution of low-level visual features to value-based decision-making. Our conclusions call for future studies using multiple item types and various measurement methods for estimating value in order to modify current theories and construct a unifying framework regarding the relationship between low-level visual features and choice.

## Introduction

People make value-based choices between different items on a daily basis. In this process, we need to construct a representation of each of the items, assign a value to it, and choose the item we prefer (Rangel et al., 2008). In order to do so, we putatively dissect the item to its low-level characteristics, such as colors (Margaret and Hubel, 1988) and spatial frequency (De Valois et al., 1982; De Valois et al., 2000). The combination of these visual features lead to higher-level representations of the objects, allowing us to identify and assign value to the item (Riesenhuber and Poggio, 1999). Several studies showed the contribution of low-level visual features to preferences of simple visual items. For color patches, more saturated and cooler colors were found to be preferred (e.g., blue was preferred over red) both in a choice task (McManus et al., 1981; Hurlbert and Ling, 2007) and in a scale rating task (Palmer and Schloss, 2010). In another choice study, subjects preferred Gabor patterns that were more symmetric (Rentschler et al., 1999).

For complex images, an important visual feature that affect preferences is the statistical property of spectral slope. Spectral slopes of nature and art images were shown to differ from slopes of other categories, such as objects or scientific charts, and this was hypothesized to relate to the aesthetic quality that one experiences when viewing art and nature (Redies, 2008; Redies et al., 2008). In addition, un-proportional enhanced energy at mid-range frequencies was found to be related to ratings of discomfort (Fernandez and Wilkins, 2008). Other visual features were also shown to influence preferences of complex images. In a study that examined natural and man-made scenes, scale-rating preferences were higher for images with higher sharpness, saturation, and contrast (Tinio and Leder, 2009). For images of snacks, items with higher luminance (Milosavljevic et al., 2012) or higher saliency (Towal et al., 2013) were more likely to be chosen in forced choice tasks. The saturation of snacks was also related to their perceived healthfulness on a scale rating task (James and Richerson, 2018). Although these studies showed the contribution of basic visual features for preferences of common objects, they each examined objects from only one category (i.e., snacks). Thus, it is difficult to depict a clear picture of the relations between basic visual features and preferences for different types of objects taken from different categories.

For complex items such as faces, the configurations between facial features are known to play an important role in face processing (for review see, Maurer et al., 2002) and face preferences (Cunningham et al., 1995; Geldart et al., 1999). The facial width to height ratio (*fWHR*) was originally proposed as an evolutionary sexual marker (being greater for males; Weston et al., 2007) and was associated with aggressive and unethical behavior (Carre and McCormick, 2008; Haselhuhn and Wong, 2011; Geniole et al., 2014, but see Kosinski, 2017). Two other studies showed that changing the distance between local face elements (between the eyes and between the eyes and nose) had a substantial effect on preferences (Searcy and Bartlett, 1996; Pallett et al., 2010). The distance between the eyes was also positively correlated with preference ratings (Cunningham, 1986). An important role for higher-level features was also shown for food items preferences. For example, the knowledge of wine prices (Plassmann et al., 2008) and of beer ingredients (Leonard et al., 2006) changed the items’ taste pleasantness ratings. It had been shown that flavors associated with high caloric foods induce greater preferences in adults, measured using a scale rating (Booth et al., 1982), and induced both greater preference ratings and actual consumption in children (Johnson et al., 1991). The literature thus holds a range of features influencing preferences, from low-level features such as luminance (Milosavljevic et al., 2012) to higher-level features such as price (Plassmann et al., 2008).

Still, it remains unclear whether the effect of low-level visual features on preferences for complex items such as faces and snacks is the same as was found for simple abstract stimuli such as patches of color. Despite ample research, the interplay between low-level visual features, item category, and methods of preference elicitation has only been discussed in reviews or meta-analyses. For example, Palmer et al. (2013) reviewed the literature that relate visual aesthetics with preferences and pointed out that different behavioral techniques, such as two-alternative forced-choice, rank order, subjective rating, and other tasks, have been used to study how visual features such as colors and spatial proprieties may contribute to the formation of preferences. Most previous studies used only one method of measurement and hence provide a limited theoretical account based on specific findings. Thus, there is a need for a unifying theory that will be able to take into account the diversity of visual features, item categories, and experimental procedures across the different studies. For instance, Palmer et al. (2013) point out that the “mere exposure” effect (Zajonc, 1968) can be used in order to explain preferences for inward over outward facing objects given that viewing inward-facing objects is much more frequent (Gardner et al., 2008). The same logic can also be applied in order to understand, for instance, why people may prefer curved visual objects (Bar and Neta, 2006). This explanation can also apply for preferences in value based tasks (e.g., Shimojo et al., 2003; Atalay et al., 2013). However, it is important to note that the “mere exposure” effect can only provide a tautological explanation to such findings and cannot offer a clear theoretical prediction for the role of low-level visual features in preference formation.

One general account that may explain preference formation is the fluency theory (Reber et al., 2004). According to this theory, the ease in which information is being processed may promote valuation. This was hypothesized to stem from the demand that object processing impose on the perceptual or cognitive system, which, in turn, translates into preferences (Palmer et al., 2013). Recently, it was proposed that fluency promotes the intensity of an existing preference, making liked items more preferred whilst disliked items even less preferred (Albrecht and Claus, 2014). Although this theory explains well how low-level features may shape preferences (Oppenheimer and Frank, 2008; Palmer et al., 2013), it is not clear if it is possible to generalize the prediction of this theory across categories. Specifically, it is not clear if low-level visual features would have any effect on preferences for faces and food items after controlling for the contribution of higher-level features, such as *Calories*, *Product weight*, and the *Price* for food items or fWHR, eye distance, and nose–eyes distance for faces. According to Fluency theory, we would expect to find relatively stable and similar relations between low-level visual features and preferences for different categories of items (assuming similar ease of processing after controlling for higher-level features), considering possible effects of other high-order, visual, and non-visual attributes, that play a role in preference formation. That is, after controlling for the influence of higher-order attributes, visual complexity alone extracts its influence on processing valuation. According to fluency theory, the way low-level visual features may promote valuation should be similar across categories. On the other hand, if different association between low-level visual features and valuation would take place as a function of stimuli category, it would be difficult to reconcile this finding based on fluency theory.

Therefore, the current study is aimed to test whether a unifying framework can account for the role of different low-level visual features of multiple item types using two measurement methods on the value-based decision-making process. Paraphrasing the well-known phrase “Beauty is in the mind of the beholder,” we ask whether beauty and valuation interact in the mind of the beholder across categories and measurement methods. Examining the role that low-level visual features play in value computations is crucial to a variety of fields, from marketing to public health, as it will increase our understanding on how subtle changes in low-level visual features may affect people’s preferences. In order to test this hypothesis, we systematically explored the influence of several low-level visual features such as color attributes (*Hue*, *Saturation*, and *Color-value*), *Sharpness*, and *Spectral-slope* on preferences for three categories of items and examined preferences using both preference ratings and binary choices. We used images of fractal-art, faces, and snack-food items as stimuli that contain different levels of higher-order complexity. Importantly, we examined the contribution of low-level features to preferences of these stimuli, while controlling for the influence of the category-specific attributes that may play a role in preference formation. Specifically, for fractal-art images, we assumed there were no higher-order attributes that affect preferences and examined only low-level visual features. However, for snacks, we added three market features (*Calories*, *Product weight*, and *Price*), and, for faces, we added three configural features (*fWHR*, *Eye distance*, and *Nose–eyes distance*). We chose this handful of features and categories as an example set of common features examined in the literature. Though many other features may be included as well, we will focus on these ones to explore the stability of effects and interplay between different features in their influence of preferences. All in all, in the present study we addressed our main research question from two perspectives: (1) the contribution of low-level visual features to preferences (measured by preference ratings) adjusted for higher-level features of different stimuli types and (2) how visual properties influence choices in a binary choice task, adjusted for the item’s preference ratings. Importantly, for robustness and generalizability, we used a large sample size collected across multiple studies in the laboratory and online and report findings after a successful pre-registered replication of the online samples. We share all our data as well as the analysis codes.

## Materials and Methods

### Participants

A total of 1,014 participants took part in the experiment (see Table 1 for sample details). All participants gave their written informed consent in accordance with the Tel Aviv University ethics committee and were paid for their participation.

**TABLE 1:** Demographics and sample details.

	Sample	Category	Age (SD)	% Female	*n*
Lab	(1)	Fractals	24.38 (3.649)	67	235
	(2)	Snacks	24.64 (3.768)	66	307

Online	(3)	Fractals	28.06 (5.397)	50	107
	(4)	Snacks	40.01 (13.042)	50	108
	(5)	Faces	42.87 (15.817)	51	119

Replication	(6)	Fractals	38.73 (13.358)	53	114
	(7)	Snacks	39.45 (13.457)	55	109
	(8)	Faces	38.42 (13.043)	46	115

Total mean			34.57 (7.064)	54 (7.207)	151.75 (71.261)

### Stimuli

We used items from three different categories. The first category was 60 fractal artwork images obtained from the internet (“Fantastic Fractals”, 2013). The second category was 60 common Israeli food snacks, all available in stores in Israel and cost up to 10 NIS (equal to ∼$2.7). All the snacks images were obtained in our lab by a professional photographer. Snacks were presented as an open package with a small portion of the snack besides the package. The third and final category was 60 faces (30 females) obtained from the sibling database (Vieira et al., 2013).

### Procedure

All experimental protocols were approved by the Tel Aviv University ethics committee. All experiments were performed in accordance with relevant guidelines and regulations.

#### Laboratory Experiments (Samples 1–2)

**FIGURE 1:**
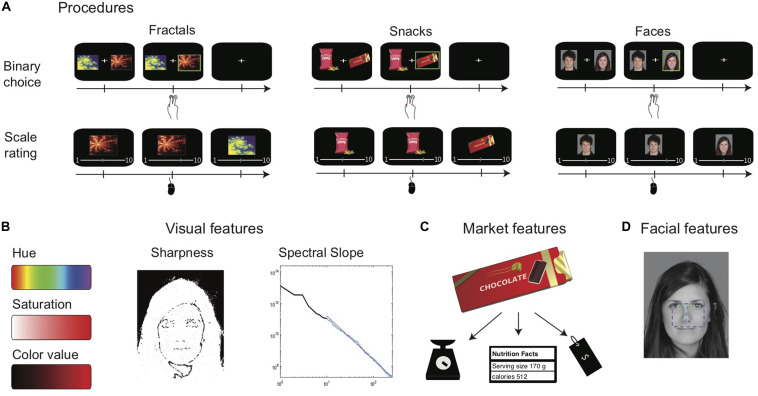
Study design. **(A)** Trial timeline in each of the two measurement methods and for the three different item types used. **(B)** The five visual features extracted for all item types. Color attributes: the color bars range from lowest **(left)** to highest **(right)** value of the feature. Sharpness: an illustration of edges extracted from a face. The Sharpness feature is set to the average of the image’s edges (without the black background). Spectral slope: the chart shows an illustration of spectral slope calculation for one item. The averages power of frequencies in black line, of it, the fitted range of 10–256 cycles is indicated in light blue, with the fitted line in dotted pink. **(C)** The three market features extracted for the food items. **(D)** The three facial features extracted for faces: *fWHR* is indicated by the purple rectangle’s aspect ratio, *Eye distance* by the green line, and *Nose–eyes distance* by the blue line. Images of faces adapted from a published open access paper (Salomon et al., 2018) and originate from a database by Vieira et al. (2013). Illustrations of two snacks are presented instead of the actual commercial snacks that were presented to participants.

We obtained preference ratings for fractals and snacks from 12 behavioral experiments (overall 342 participants) conducted in the lab [of which eight experiments had both fractals and snacks ratings (*n* = 200), one with only fractals (*n* = 35) and three with only snacks ratings (*n* = 107)]. All these experiments had a standard rating procedure before any exposure to the rated items occurred. Given that the rating task was similar across these 12 behavioral experiments, we examined the interaction between samples and the effects of interests with multiple linear regression models and found none of them to be significant (all *p*’s > 0.05). We thus combined all preference ratings in the laboratory as if they were collected from a large joint sample, one for fractals (sample 1) and the other for snacks (sample 2; see Table 1 for details). In the lab experiments, participants rated their preferences for the fractals using a continuous numerical scale in which they indicated how much they liked each item from 1 to 10 (see Figure 1A). For the snack foods, participants indicated their willingness to pay (WTP) for each food item using the incentive-compatible Becker–DeGroot–Marschak auction (Becker et al., 1964; see also Salomon et al., 2018 for detailed procedure, as explained in the section “Auction Procedure for Snack-Food Items”), with a continuous price scale of 1–10 NIS. We used the WTP task, as it is considered a task that elicits the participant’s preference ratings for the food snacks. Participants used a mouse to indicate their preference on a continuous number scale. The procedure was self-paced, and each item was presented once, resulting in 60 trials per participant for each category. Faces were not rated in lab experiments. There were no binary choices in the lab experiments.

#### Online Experiments (Samples 3–5)

To replicate the lab preference ratings and explore the effects of visual features on binary choice tasks, we obtained choices and preference ratings in three online experiments (for fractals, snacks, and faces). Note that, for snacks, the procedure was a scaled preference rating and not the incentive compatible BDM since it was performed online. Overall, 334 participants took part in the online experiments, of which 107, 108, and 119 participated in the fractals, snacks, and faces experiment, respectively. All online experiments were conducted via an Israeli online website^1^ that specializes in conducting online experiments. Each participant performed the binary choice task followed by the preference rating procedure (see Figure 1A) of one of the categories (fractals, snacks, or faces). In the binary choice task, ∼14% of all possible binary choice combinations (60 × 59/2, resulting in 240 trials per participant) were randomly selected and presented for each participant. On each trial, participants indicated which of the two items they preferred by pressing the keyboard. Each choice was presented for 2.5 s, followed by a 1 s fixation cross presented at the center of the screen. The preference rating for all categories (fractals, snacks, and faces) was obtained via the non-incentive scale rating procedure, which was identical to the lab preference rating procedure, described above.

#### Replication Experiments (Samples 6–8)

To obtain a full replication of our data, we pre-registered our data acquired from samples 1–5 and performed an identical replication of the above online experiments^2^. Overall, 338 participants took part in the replication online experiments, of which 114, 109, and 115 participated in the fractals, snacks, and faces experiments, respectively.

### Feature Analyses

#### Visual Features

We extracted five visual features for each item (see Figure 1B) using Matlab (Mathworks, Inc. Natick, MA, United States, SCR: 001622): *Hue*, *Saturation*, and *Color value*, the color attributes according to the HSV color-map (Joblove and Greenberg, 1978), were calculated as the mean attribute of the item’s image. *Sharpness* was calculated as the mean image gradient (Ferzli and Karam, 2009) using the Sobel–Feldman operator (Sobel and Feldman, 1968). *Spectral-slope* was calculated according to Mather (2014): we converted all images to gray-scale, resized them with bicubic interpolation such that the short dimension is 512 pixels, and extracted 512 × 512 pixels from the center of the image for further analysis. We then performed Fourier transformation to convert the image to the frequency domain and calculated the rotational average of the power spectrum. Finally, we fitted a least square linear line on the rotational averages between 10 and 256 cycles (this range is used in order to avoid artifacts from extreme low or high frequencies). We defined the *Spectral slope* of the image as the slope of the least square line.

In addition, we acquired the following category specific features for faces and snacks:

#### Facial Features

We extracted three facial features for faces (see Figure 1D) using the Viola–Jones algorithm (Viola and Jones, 2001) using Matlab: (1) *Eye distance* (the distance between the two eyes normalized by face size); (2) *fWHR*; and (3) *Nose–eyes distance* (the distance between the bottom of the nose and the center of the two eyes, normalized by face size).

#### Market Features

We collected three market features for each of the snacks (see Figure 1C): (1) *Price*; (2) *Product weight* (in grams); and (3) *Calories* (per 100 g). We extracted the information from the labeling on the snack’s package and from the Internet. Detailed correlation matrices of all features are reported in Supplementary Figure S1.

### Behavioral Analyses

Both the WTP scores and the preference ratings were obtained on continuous scales with a mean of 3.48 (2.589 SDs) for fractals and 3.54 (2.531 SDs) for snacks in the lab samples, 4.64 (2.532 SDs) for fractals, 5.32 (2.760 SDs) for snacks and 4.82 (2.071 SDs) for faces in the online samples, and 4.78 (2.537 SDs) for fractals, 5.43 (2.813 SDs) for snacks, and 4.83 (2.249 SDs) for faces in the replication samples. All WTP and ratings were z-scored separately for each participant to remove variance between participants resulting from them using different ranges of the scale. All the extracted values of the features (*visual*, *market*, and *facial* features) were also z-scored to enable a direct comparison of regression coefficients. We removed from further analysis items with feature values exceeding 3 standard deviations (SDs) away from the mean (1 fractal, 2 snacks, and 1 face). We excluded trials with reaction times exceeding 3 SDs away from the mean (calculated within task and within participant) or trials with no response. Overall, we removed an average of 3.23% (1.455 SDs) of trials per participant across all samples. In addition, we removed participants with more than 30% excluded trials in either ratings or choice data and participants with extreme intransitivity in their binary choices (exceeding 3 SDs away from the mean of the sample’s transitivity scores). The transitivity score was calculated as the SD of Colley Matrix algorithm (Colley, 2002), as was also performed in previous studies in our lab (Salomon et al., 2018). We removed 20 participants in the online and replication samples (between two and five participants for each sample), and we concluded with 1,014 valid participants overall in all samples. No participants were removed from the lab samples. In the binary choice task, right and left displays were randomly assigned on each trial. Proportions of choosing the left item were 0.496 for fractals, 0.472 for snacks, and 0.495 for faces in the online samples and 0.498 for fractals, 0.481 for snacks, and 0.496 for faces in the replication samples. We performed all data analyses in R (version 3.3.2. SCR: 001905).

### Statistical Analyses

#### Is There a Linear Relationship Between Low-Level Visual Features and Category-Specific Features With Preference Ratings?

To examine the influence of low-level visual features and category-specific features (market and facial features) on preference ratings, we fitted for each category a linear mixed-effects regression model (see Supplementary Model S1 for detailed formulas) with a random-intercept and random slopes. That is, we allowed the intercept and the slope coefficients of each of the features (visual and category-specific features) to vary across participants. Ratings served as the dependent variable and the different features as fixed and random independent variables. We fitted this model separately for each of the lab (samples 1–2), online (samples 3–5), and replication (samples 6–8) samples. For each of the samples, we entered all features together to the regression model. Thus, the results reflect the unique contribution of each feature adjusted for all other features.

#### Do Low-Level Visual Features and Category-Specific Features Affect Binary Choices That Have Been Adjusted for Preference Ratings?

To examine the influence of the different features on choices, adjusted for preference ratings, we first determined the preference ratings of each item for each participant using their ratings in the scale rating task. We then calculated the ratings value difference between the two items in every choice option (hereafter *delta ratings*, e.g., Milosavljevic et al., 2012). We chose to account for rating value differences in each choice option since they are expected to have a strong influence on choice. By entering *delta ratings* into the regression models, we allowed the exploration of more subtle effects of visual features on choices.

Furthermore, for each of the features (visual and category-specific features) we extracted the score difference between the two items (left item minus right item) in every choice option (hereafter *delta feature*, e.g., *delta Hue*, *delta Price*, etc.). For each of the samples, we entered the delta *ratings* feature with all delta visual and delta category-specific features together to the regression model. We fitted for each category a mixed-effects logistic regression (see Supplementary Model S2 for detailed formulas). We fitted a random-intercept and random-slope model with choices as the dependent variable and *delta ratings* and all *delta features* (the delta of visual, market, and facial features) as fixed independent variables. We allowed for the intercept and slope of *delta ratings* to vary across participants. We fitted this model separately for each of the online (samples 3–5) and replication (samples 6–8) samples.

Data and code sharing: all data and analyses codes are available at https://osf.io/zmp49/?view_only=8c65101a29f14140b771aa87bcd91106.

## Results

The current study is composed of many samples with numerous possible effects, and we thus report below the summary of effects. Detailed description of all model results with effect sizes and confidence intervals are reported in Supplementary Tables S1, S2. As mentioned above in the methods, lab experiments consisted of only preference ratings (or BDM for snacks), whilst in the online and replication experiments, participants performed the binary choice task and then the preference rating task for one of the categories.

### Is There a Linear Relationship for Low-Level Visual Features and Category-Specific Features With Preference Ratings?

**FIGURE 2:**
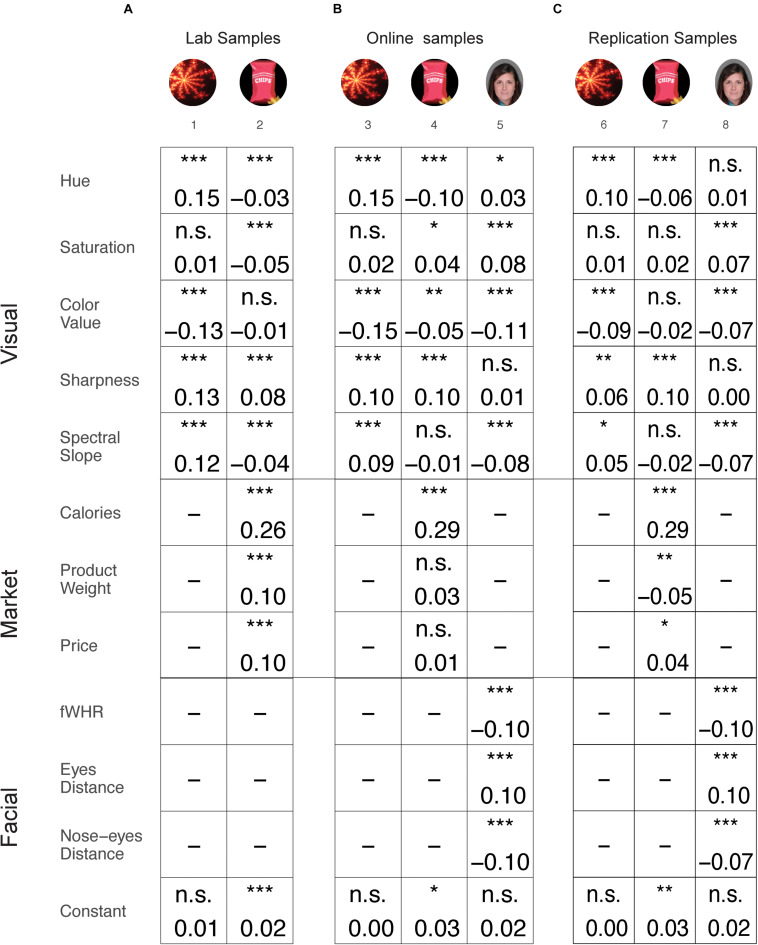
Results summary of the mixed linear regression models for ratings. Effects presented separately for lab **(A)**, online **(B)**, and replication **(C)** samples. Each column represents a different sample and each row represents a different feature. The value in the square indicates the coefficient value for the current feature in the current samples’ model. Black text indicates that the current feature was substantial across all samples in the category. Gray text indicates that one or more samples of this category were not significant, thus the effect was not stable across all samples. *p < 0.05; **p < 0.01; ***p < 0.001. Images of faces adapted from a published open access paper (Salomon et al., 2018) and originate from a database by Vieira et al. (2013).

In ratings data obtained from the experiments conducted in the lab, we found that each of the visual features had a different influence on preference ratings and, in some cases, an opposite effect, depending on the items’ category (Figure 2A). Specifically, *Hue* had a positive effect on preference ratings of fractals whilst a negative effect on snacks. *Saturation* had a negative effect only on snacks, and *Color-value* had a negative effect only on fractals. *Sharpness*, however, had a positive effect for both fractals and snacks, and *Spectral-slope* had a positive effect for fractals whilst a negative effect for snacks.

In order to examine the robustness of these results we repeated the lab experiments on an online cohort of participants. This complex pattern of relations between visual features and item category and their influence on preference ratings was mostly replicated in the online experiments (Figure 2B). That is, *Hue* had a positive effect on fractals (*sample 3*) and a negative effect on snacks (*sample 4*). *Color value* had a negative effect on fractals (*sample 3*), *Sharpness* had a positive effect on both fractals and snacks (*samples 3 and 4*), and *Spectral slope* had a positive effect on fractals (*sample 3*). The negative effects of *Saturation* and of *Spectral slope* on snacks in the lab data were not replicated in the online samples. In addition, we found a positive effect for Saturation and a negative effect for *Color value* on snacks, which were absent in the lab samples.

In addition to the samples of fractals and snacks, in the online experiments, we collected data of preference ratings for faces. Similar to the fractals and snacks, we found that there was an effect of visual features on preference ratings for faces. However, in general, these were different features, and the direction of influence was different compared to the effects we found for fractals and snacks. That is, *Hue* and *Saturation* had a positive effect and *Color value* and *Spectral slope* a negative effect on preference ratings for faces (*sample 5*). These results further support our finding that the effect of visual features on preference ratings is category specific.

We then examined the replication results of our pre-registered samples, which served as a second replication of the effects of visual features on preference ratings for fractals and snacks (following the lab and online samples) and a replication for faces (following the online sample). Importantly, we replicated the complex pattern we obtained in the lab and online samples (Figure 2C). Specifically, *Hue* had a positive effect on fractals (*sample 6, similar to samples 1 and 3*) and a negative effect on snacks (*sample 7, similar in samples 2 and 4*). *Color value* had a negative effect on fractals (*sample 6, similar in samples 1 and 3*), *Sharpness* had a positive effect on fractals and on snacks (*samples 6 and 7, similar to samples 1 and 2 as well as 3 and 4*), and *Spectral slope* had a positive effect on fractals (*sample 6, similar in samples 1 and 3*). For faces, *Saturation* had a positive effect, and *Color value* and *Spectral slope* had a negative effect (*sample 8, similar to sample 5*). The negative effect for *Saturation* on snacks in the lab data (*sample 2*), which was reversed in the online sample (*sample 4*), was not significant in the replication samples (*sample 7*). The effects that were found in the online samples for *Color value* on snacks (*sample 4*) and for *Hue* on faces (*sample 5*) were not replicated in the replication samples (*samples 7 and 8*).

In order to investigate the role of basic visual features adjusted for the effects of higher-level features, we added market features for snacks and facial features for faces to the regression models, alongside the visual features. The effects of the higher-level features were examined and replicated to some extent. For snacks, we found a positive effect for *Calories, Product weight*, and *Price* in the lab sample (*sample 2*). The effect of *Calories* on ratings was replicated first in the online sample (*sample 4*) and again in the replication sample after pre-registration (*sample 7*). However, the effects of *Product weight* and *Price* were not replicated in the online sample (*sample 4*). In the replication sample (*sample 7*), the effect of *Price* was replicated, but the effect of *Product weight* was reversed. For faces, we found a negative effect for *fWHR*, a negative effect for *Eye distance* and a positive effect for *Nose–eyes distance* in the online sample (*sample 5*). These effects for faces were fully replicated in the replication sample after pre-registration (*sample 8*).

Overall, the category-dependent pattern for the influence of visual features on preference ratings was stable across independent samples and experimental settings (lab vs. online experiments). For detailed results of the regression models, see Supplementary Table S1.

### Do Low-Level Visual Features and Category-Specific Features Affect Binary Choices Adjusted for Preference Ratings?

**FIGURE 3:**
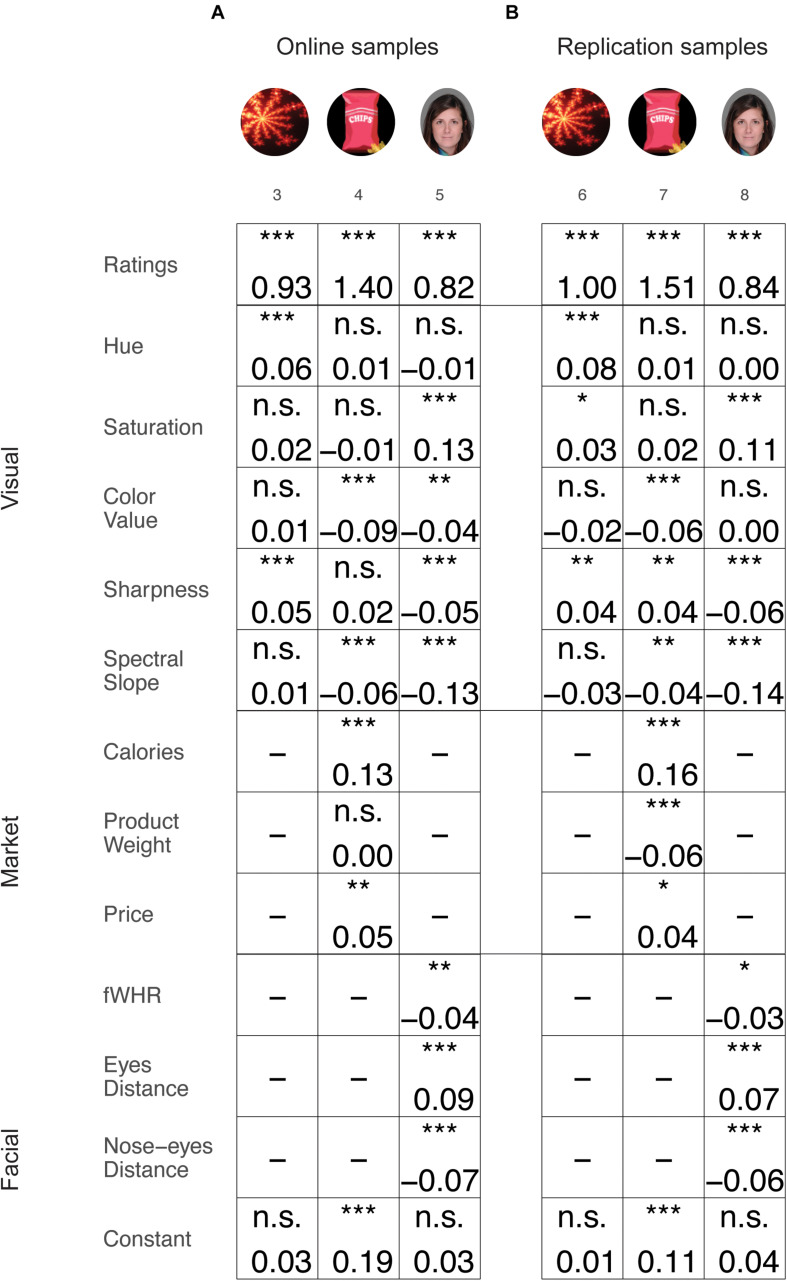
Results of the mixed logistic regression models for choices. Effects presented separately for online **(A)** and replication **(B)** samples. Each column represents different samples and each row represent different features. The value in the square indicates the coefficient value for the current feature in the current samples’ model. Black text indicates that the current feature was substantial across all samples of this category. Gray text indicates that one or more samples of this category were not significant, and the effect was thus not stable across samples. *p < 0.05; **p < 0.01; ***p < 0.001. Images of faces adapted from a published open access paper (Salomon et al., 2018) and originate from a database by Vieira et al. (2013).

We next examined the influence of low-level visual features and category-specific features on actual choices after we controlled for their value, as indicated in their preference ratings. Similarly to the preference ratings results, the effect of the various features on choices was category specific. That is, each feature affected choice differently and this effect depended on the specific items’ category (Figure 3). Note, that these effects are after controlling for the higher-level features in the model (facial and market) and were mostly replicated in the independent replication samples (*samples 6, 7, and 8*). It is important to emphasize that the effects of the various features on choice (described in Figure 3) exist after adjusting for the items’ preference ratings, as the ratings of each item were included in the regression model. That is, visual features can impact choices, regardless of the items’ ratings value.

Particularly, participants tended to choose items with higher *Hue* in fractals (*sample 3*), higher *Saturation* in faces (*sample 5*), lower *Color value* in snacks and in faces (*samples 4 and 5*), higher *Sharpness* in fractals (*sample 3*) but lower *Sharpness* in faces (*sample 5*), and lower *Spectral slope* in snacks and faces (*sample 4 and 5*). For the category-specific features, participants tended to choose items with higher *Calories* and higher *Price* in snacks (*sample 4*) and higher *Eye distance* and lower *fWHR* and *Nose–eyes distance* in faces (*sample 5*). All these effects were then replicated in the replication samples (*sample 6* for fractals, *7* for snacks, and *8* for faces), except for the effect for *Color value* in faces, which was not significant (*sample 8*). In addition, we found positive effects for *Saturation* on fractals (*sample 6*), a positive effect for *Sharpness*, and a negative effect for *Product weight* on snacks (*sample 7*), which was not found in the online sample (*samples 3 and 4*). These results indicate a pattern, by which different visual features influence choices between items, in a replicated manner within the same category but not across categories. For detailed results of the regression models, see Supplementary Table S2.

### Effects Across Tasks

**FIGURE 4:**
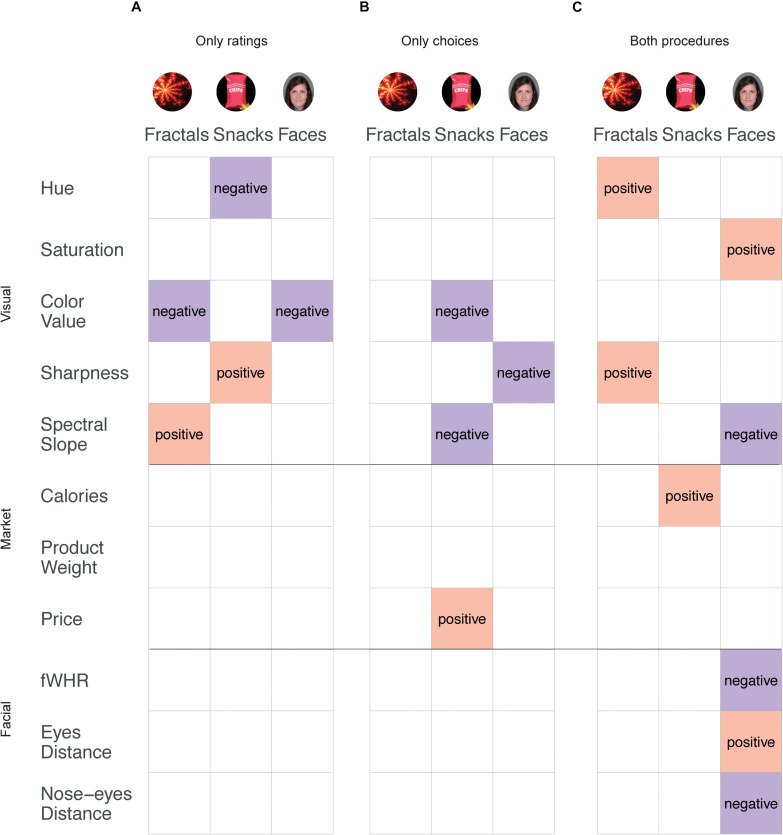
Summary of results from both ratings and choice data. All effects shown here were stable across all samples for each category, for only ratings **(A)**, only choices **(B)**, or both ratings and choices **(C)**. Columns represent categories and rows represent features. The color indicates the direction of the effect (pink: positive, purple: negative). Images of faces adapted from a published open access paper (Salomon et al., 2018) and originate from a database by Vieira et al. (2013).

We demonstrate a complex pattern of the effects of basic visual features on preference ratings and binary choices. Figure 4 shows a summary of the effects that were replicated, obtained only in preference rating (Figure 4A), only in choices (Figure 4B), and in both procedures (Figure 4C). Note that we address the latter options as exclusive, meaning an effect that was observed in both procedures will not appear in the “only ratings” or “only choices” options. There were several effects that were similar across both task procedures. For the basic visual features, *Hue* and *Sharpness* had a positive effect on fractals, *Saturation* had a positive effect on faces, and *Spectral slope* had a negative effect on faces. For the higher-level features, *Eye distance* (Facial) had a positive effect, while *fWHR* and *Nose–eyes distance* had a negative effect on faces. *Calories* (Market) had a positive effect on snacks. Hence, these effects are stable across independent samples (including a replication of pre-registered samples) and could be generalized across measurement procedures.

In contrast, there were several effects that were replicated only in one task procedure but not in the other. In the preference ratings task, *Color value* had a negative effect for fractals and faces, *Hue* had a negative effect on snacks, *Sharpness* had a positive effect on snacks, and *Spectral slope* had a positive effect on fractals.

On the other hand, in the choice task, *Sharpness* had a negative effect on faces, while *Color value* and *Spectral slope* had a negative effect on snacks. Note, that there was no clear and replicated effect on fractals in the choice task that was absent in the ratings task. For *the higher-level* features, only market features replicated solely in the choice task, as *Price* had a positive effect on choices.

These results further emphasize the uniqueness of the effects, by which different visual features influence preferences or choices between items, in a replicated manner within the same category, but not across categories or across measurement procedures. We note that when testing the role of visual features on binary choice we accounted for the subjective ratings of each item. Therefore, the influence of a feature on ratings is statistically accounted for when we examine the effect of that feature on binary choices. The differences between measurement tools thus imply that visual features influence each measurement differently.

## Discussion

Most studies thus far have shown the influence of isolated visual properties, such as contrast or hue, on specific aesthetic items (e.g., paintings, abstract images, etc.; Palmer et al., 2013). However, an investigation of isolated types of items does not provide the possibility to examine the contribution and interactions of different features on different types in parallel and of different measurement methods. In the current study, we tested for the first time in one study, the influence of low-level visual features on preferences of fractal art images, faces and snack-food items, using both ratings and binary choices. We show that low-level visual features contribute to preferences differently for each stimulus type and measurement method. We focused on five basic visual features that have a key role in low level visual processing: the three main color features of *Hue*, *Saturation*, and *Color value* (Margaret and Hubel, 1988), *Sharpness* (Ferzli and Karam, 2009), and *Spectral Slope* (Burton and Moorhead, 1987; Field, 1987). For colors, most studies that examined color preferences on simple color patches stimuli had shown a general preference toward cooler and brighter colors (i.e., higher *Hue*, *Saturation*, and *Color value*; McManus et al., 1981; Hurlbert and Ling, 2007; Palmer and Schloss, 2010). However, we show that for complex items, this tendency was replicated only for the *Hue* of fractals and *Saturation* of faces. Namely, participants preferred fractals with higher *Hue* (but not faces or snacks) and faces with higher *Saturation* (but not fractals or snacks) in both rankings and choices. Moreover, we found the opposite effect for *Color value*, which showed a negative effect on rankings of fractals and faces and on choices of snacks. For snacks, we also found an opposite effect for *Hue* on ratings, showing higher preferences for lower hues. This trend may corresponds with a previous study showing that snacks packaging with lower hues are being perceived as healthier (James and Richerson, 2018), but further studies are needed to support this interpretation.

For complex images, in line with a study where preference for greater *sharpness* was shown (Tinio and Leder, 2009), we found preference for greater sharpness for fractals in both ratings and choices and for snacks only in ratings. For faces, however, we found the opposite preference toward lower sharpness in choices and no effect on ratings. For the *Spectral Slope* of the images, studies had shown differences in its mean and variance between categories (Redies et al., 2008; Mather, 2014), and it was hypothesized to be related to the aesthetic perception of the images (Redies, 2008). Yet, it is not clear if and in what way would differences in the *Spectral Slope* of images within a certain category relate to preferences. Here, we found a negative effect for *Spectral Slope* on faces in both procedures and for snacks only in choices, but a positive effect for fractals only in ratings. Based on the current literature, it is difficult to find definitive reasons for why a specific feature contributed to one category over the other. This is one of the main conclusions of our study: when testing only a certain feature using a certain methodology on a specific stimulus type (as was mostly done in previous studies), it is hard to generalize across domains and features.

Furthermore, our results provide insights regarding the effects that higher-level features have on preferences and choices. For snacks, we found that participants like items with higher *Calories*, as indicated both in their ratings and choices, which is in line with other studies that showed higher preferences for high-calorie foods (Booth et al., 1982; Johnson et al., 1991). In addition, choices were influenced by the snacks’ *Price*, in accordance with studies showing the effect of price expectations on preferences (Leonard et al., 2006; Uher et al., 2006). This is in line with imaging studies examining the neural correlates of these elements, such as choices vs. ratings (Shenhav and Karmarkar, 2019) and preferences vs. *Price* (Knutson et al., 2007). Still, the lack of effect of *Price* on preference ratings is interesting and calls for further investigation. Note, that we collected the snack’s higher-level features from information detailed on the packaging or the Internet. However, there might be a substantial difference between the actual features (i.e., the number of *Calories* in a snack, the *Product weight*) to the perceived features (i.e., the number of *Calories* the participant thinks are in the snack and how big the snack is in the eye of the participant). In addition, it is not clear how such higher-level features contribute to the perception and identification of the items or contribute directly to their preferences. Future studies may wish to examine the variations of actual versus perceived levels of such features in their effects on preferences.

For faces, in line with previous work, participants preferred faces with greater distance between the eyes and nose in both ratings and choices (Cunningham, 1986). Additionally, participants preferred faces with lower nose to eyes distance and smaller *fWHR* in both procedures. The effects of these two features were inconclusive in previous studies. The nose to eyes distance was shown to correlate with preferences in an inverted U-shape (Pallett et al., 2010), whilst the Mid-face range (corresponding with this feature) had no correlation with preference (Cunningham, 1986). For *fWHR*, a recent large sample study claimed for a null effect for this feature (Kosinski, 2017). Our study thus sheds light on the interaction between facial features and preferences, however, further studies are required to determine the exact role of these features on preferences.

We chose to present only two regression models in the main results: showing the relation of features on scale ratings and showing the effects of features on binary choices, while taking into account the scale ratings (i.e., entering them into the statistical model). We chose to conduct the analysis in such a manner following a seminal paper, which examined the effects of saliency on binary choices, after entering scale rating to the model (Milosavljevic et al., 2012). Yet, there are many different analyses that could have been done with our current data to explore the relation between features and preferences. For example, we show additional models in the supplementary: one for explaining preference ratings after entering the binary choices to the model (see Supplementary Figure S2) and the second for explaining binary choices without entering preference rating to the model (see Supplementary Figure S3). It is not surprising that different analysis models lead to slightly different results (Botvinik-nezer et al., 2019). Indeed, we suggest that this strengthens our main conclusion that multiple items types and categories should be tested together. These additional analyses emphasize the need for a more robust approach, given that the kinds of “isolated” effects reported thus far should be considered with caution as they were dependent on the category, task, and analysis used in each study.

In the current study, we focused on a selected number of categories and features we found to be abundant in the literature. Future studies are needed to explore additional categories and features that were beyond the scope of this study. For instance, how would higher level features, such as *Price* and *Product weight*, influence different categories such as supply goods or restaurants meals in addition to snacks? How would the complexity of different features influence their interplay with each other and their influence on preferences?

As we suggested in the introduction, according to fluency theory (Reber et al., 2004; see also Palmer et al., 2013), the ease in which information is being processed may promote valuation. That is, people prefer displays or objects they can perceive easily and impose less demand on the perceptual or cognitive system. Contrary to the prediction of fluency theory, that is, to find relatively stable and similar relations between low-level visual features and preferences for different categories of items, we found a unique pattern per category that relates low-level visual features and preferences. Therefore, our results suggest that category type serves as a strong moderator between low-level visual features and valuation. Moreover, it is important to note that our design controlled for the influence of higher-order, category-specific attributes on valuation. One way to reconcile this finding with fluency theory is to address other low-level visual features that may extract their influence on valuation such as line orientation, complexity, and symmetry, contour curvature and compositional biases (for more details see Palmer et al., 2013) that were not considered in the current study. It is possible that such confounding variables may also influence ease of processing. Nonetheless, we choose to emphasize specific features (*Hue*, *Saturation*, *Color value*, *Sharpness*, and *Spectral Slope*) that have been widely investigated separately in previous work (Burton and Moorhead, 1987; Margaret and Hubel, 1988; Ferzli and Karam, 2009) and are taken as the corner stone of the research in aesthetics preference. Further studies are needed in order to rule out the influence of other visual factors.

Another possible explanation to our findings is that each category involves a different level of abstraction. The current design helps us to revel the unique contribution of low-level features on valuation while controlling for the contribution of higher-level features. However, it is possible that the processing of more complex stimuli, such as face and food items, require that more controlled, top-down processing would take place. Different models in cognitive and system neuroscience proposed that visual analysis start with parallel extraction of different spatial frequencies of a given image. These models suggest that the time course of visual processing follows a predominately “coarse-to-fine” processing strategy (Bullier, 2001; Hochstein and Ahissar, 2002; Bar et al., 2006; Hegdé, 2008). For example, according to Bar and colleagues, early visual magnocellular information is projected to the orbitofrontal cortex (OFC) where it triggers top-down facilitation of object recognition by generating associations that direct the predictions, which are crucial in order to constrain bottom-up processes in favor of a small set of relevant possibilities (Bar et al., 2006). It is possible that top-down processing is category specific; for example, fractals and unfamiliar faces do not convey the same information as well-known snacks. This difference may affect how top-down processing directs and influences the way lower-level visual attributes may be manifested and influence valuation processes. Future research can explore how top-down processing is category-specific by using other types of stimuli such as artificial vs. naturalistic, curved vs. sharp (Bar and Neta, 2006), abstract shapes vs. recognizable objects (Silvia and Barona, 2009), and objects with different subjective valences.

Recent work suggests that common principles underlie both subjective valuation and sensory perception (Polanía et al., 2019). We suggest that value representation complies with this very basic feature of the brain’s ability to process information mentioned above, i.e., the ability to follow a “coarse-to-fine” strategy. Our results may suggest that subjective valuation described as top-down narrowing of bottom-up processing is category dependent. That is, it is possible that during subjective valuation, the type of stimulus at hand, emphasizes the related features that are more associated and relevant for narrowing the bottom-up process. This suggestion is in line with Bar’s predicted hypothesis by which top-down processing aids in generating associations that direct the predictions, which are crucial to constrain bottom-up processes in favor of a small set of relevant possibilities (Bar and Neta, 2006). These associations may be specific features of the stimuli and are category dependent. Using “coarse-to-fine” models may offer a parsimonious explanation to different observed effects of choice variability, biases, or diversions from rationality. These models have plausible assumptions regarding the limited-capacity nature of our biological system together with a clear neurobiological and cognitive basis.

Following the replication crisis in different scientific fields (Prinz et al., 2011; Nave et al., 2015; Lithgow et al., 2017), the research community has become committed to producing reproducible science, using larger sample sizes, pre-registrations, sharing open codes, and data (Nosek et al., 2012, 2015). Therefore, here we share all our data, analysis, and task codes. Furthermore, a major part of our findings has been pre-registered prior to collecting additional data, and we were able to replicate them in three new samples. This provides further robustness and generalizability to our results. We show that the effects of visual features on preferences are stable across samples and are not similar across categories. Future studies could examine the generalizability of the visual features we used on other stimuli within each category of items to examine whether the effects obtained in the current study are stimuli specific.

To conclude, we offer for the first time an elaborate testing of multiple visual features on multiple categories with several measurement tools to show that the influence of low level visual features is complex and specific to the item category tested and the way items’ value was estimated (either by preference ratings or choice). Moreover, we demonstrated that low-level features affect preference ratings and also influence choices even after controlling for preference ratings, showing that these effects are sustainable and independent of items’ value. Our results emphasize the importance of examining multiple features and categories, rather than deducing the influence of a certain feature on preference for a certain category. We chose these item categories and specific features as they are commonly used by us and others in value-based decision-making experiments and in perceptual visual experiments and thus, we aimed to test the commonality and differences in the effects between them. Naturally, there are many other possible features and categories that we did not examine that could have been tested. Importantly, we were able to replicate almost all effects of the low-level visual features that we found in the current study, demonstrating that these effects are stable and could be generalized across samples. This exemplifies the importance of pre-registration and testing our results using independent samples in order to obtain robust conclusions. Future studies are suggested to follow this approach of testing multiple features, on multiple items in different settings potentially on the same participants, to take into account as many features as possible to be able to shed light on the long-lasting question posed by Fechner (1871) regarding the influence of visual features on preferences.

## Data Availability Statement

The datasets generated for this study are available on request to the corresponding author.

## Ethics Statement

The studies involving human participants were reviewed and approved by Tel Aviv University Ethics Committee. The patients/participants provided their written informed consent to participate in this study.

## Author Contributions

SO designed the experiments, collected and analyzed the data, and wrote the manuscript. TSe assisted with design of the experiments, data analysis, and contributed to write-up of the manuscript. DL contributed to the design of the experiments, data analysis, and manuscript write-up. TSc designed the experiments, assisted with data analysis, and wrote the manuscript with SO. All authors actively contributed to the writing process of the manuscript.

## Conflict of Interest

The authors declare that the research was conducted in the absence of any commercial or financial relationships that could be construed as a potential conflict of interest.

## Footnotes

1 https://www.midgampanel.com/

2 https://osf.io/zmp49/?view_only=8c65101a29f14140b771aa87bcd91106
