# Supplementary material for: Item Features Interact With Item Category in Their Influence on Preferences
Source: Front Psychol. 2020 Jul 23;11:988. doi: 10.3389/fpsyg.2020.00988 (PMC7391002; doi:10.3389/fpsyg.2020.00988)
Supplement: Supplementary file 1 [file Data_Sheet_1.pdf]

S1 Fig. Pearson correlation matrices of the feature's regressors. Results for fractals (a), snacks (b) and faces (c), ranging from lowest (blue) to highest (red) correlation value. Square brackets represent confidence intervals of α=0.05.

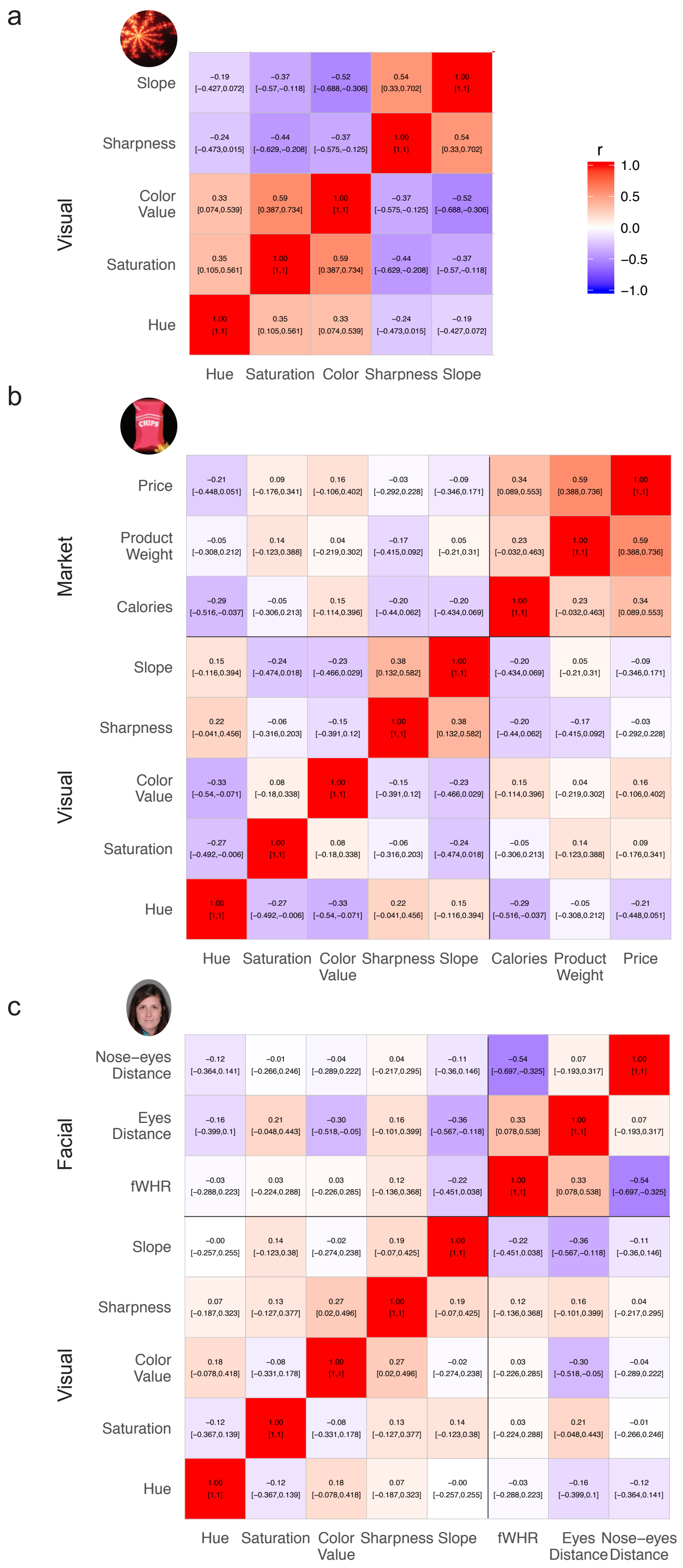

S2 Fig. Results summary of the mixed linear regression models for ratings. Effects presented separately for online (a, b) and replication (c, d) samples. (a, c) examine the effects of the different features on ratings, after accounting for preferences as indicated by a binary choices task. We calculated preferences from binary choices using the Colley Matrix Algorithm (Colley 2002, see also: Salomon et al. 2018 for detailed procedure, as explained in the section 'Binary ranking for non-consumables items'). Each column represents different samples and each row represent different features. The color and value of the square indicates the coefficient value for the current feature in the current samples' model, from high (red) to low (blue). Black text indicates that the current feature was substantial across all samples of this category. Gray text indicates that one or more samples of this category were not significant, thus the effect was not stable across samples. * p<0.05; ** p<0.01; *** p<0.001. Images of faces adapted from a published open access paper (Salomon et al. 2018), and originate from a database by Vieira, Bottino and Laurentini (2013).

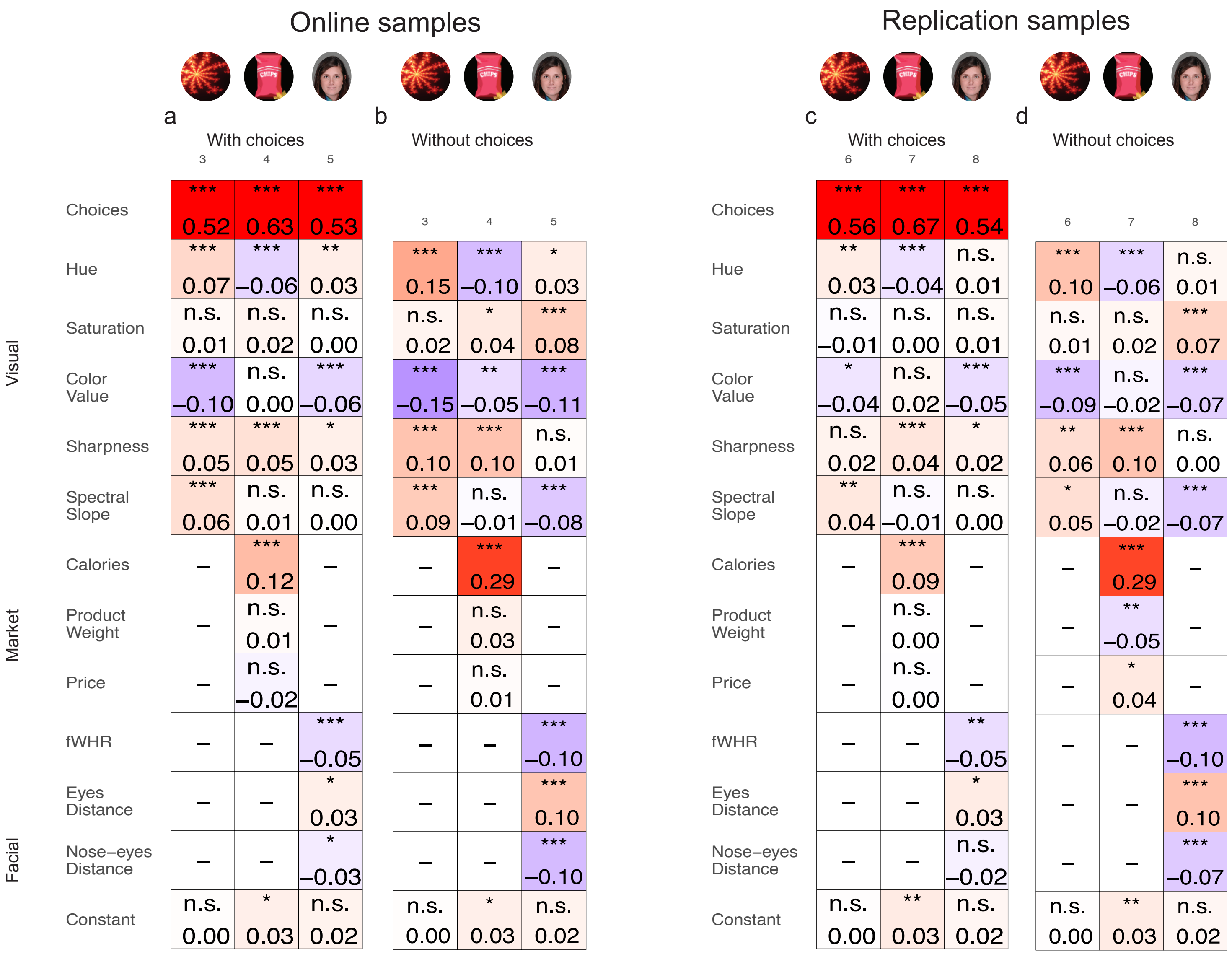

S3 Fig. Results of the mixed logistic regression models for choices. Effects presented separately for online (a, b) and replication (c, d) samples. (a, c) examine the effects of the different features on binary choices, after accounting for preferences as indicated by a scale rating task. Each column represents different samples and each row represent different features. The color and value of the square indicates the coefficient value for the current feature in the current samples' model, from high (red) to low (blue). Black text indicates that the current feature was substantial across all samples of this category. Gray text indicates that one or more samples of this category were not significant, thus the effect was not stable across samples. * p<0.05; ** p<0.01; *** p<0.001. Images of faces adapted from a published open access paper (Salomon et al. 2018), and originate from a database by Vieira, Bottino and Laurentini (2013).

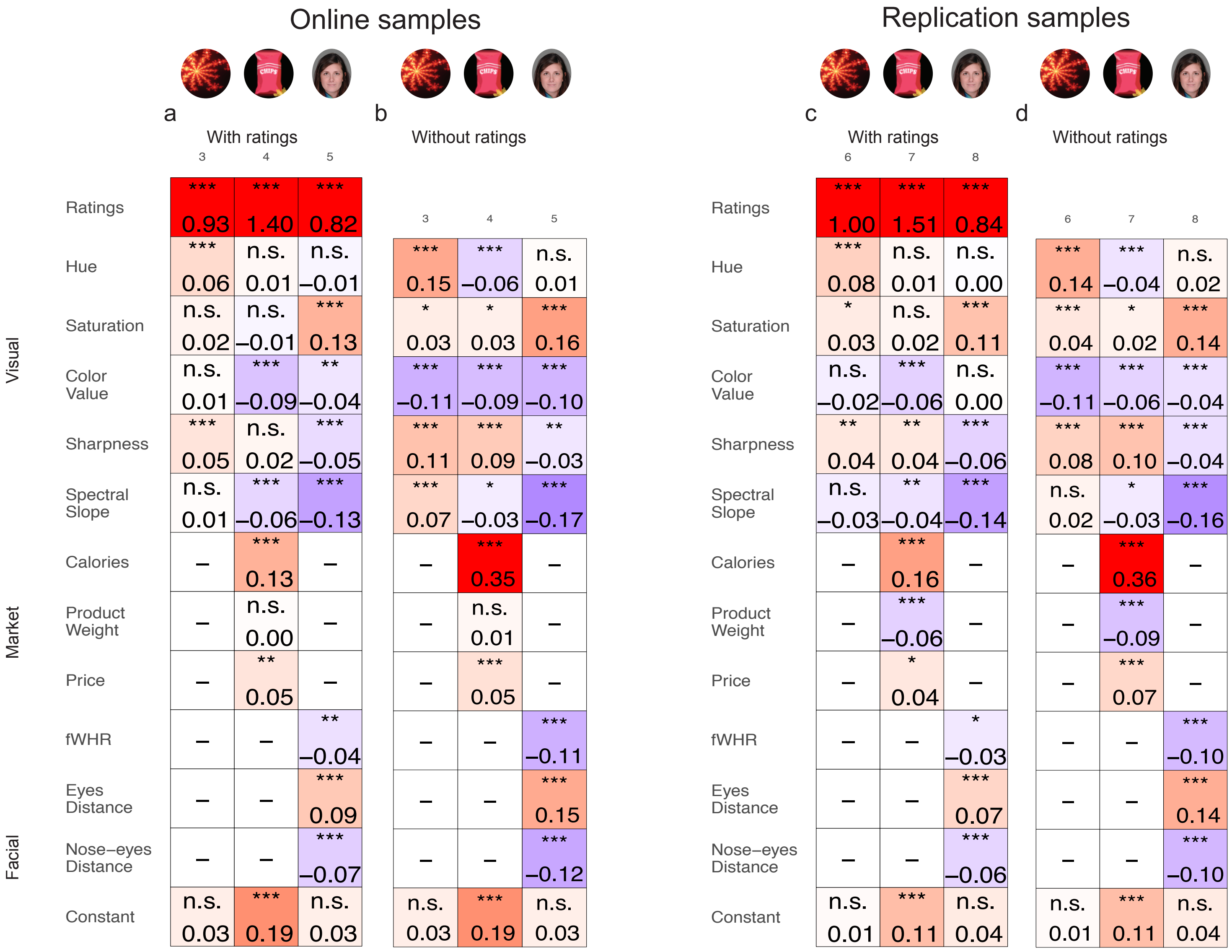

S1 Model. Equations of model 1 linear regressions. We fitted a linear mixed-effects regression models to explain preference ratings. All regressors (i.e. Visual for fractals, Visual and Market for snacks and Visual and Facial for faces), served as fixed and random factors, with uncorrelated random intercept and slope (Bates et al. 2014). All models were calculated in R (version 3.3.2), with the lme4 package (version 1.1-14). The R code of model 1 equations for (a) fractals, (b) snacks and (c) faces is presented below. p=participants.

a. Fractals: *lmer (data=RankData, Ratings ~ Hue + Saturation + Color value + Sharpness + Spectral Slope + (Hue||p) + (Saturation||p) + (Color value||p) + (Sharpness||p) + (Spectral Slope||p))*

b. Snacks*: lmer (data=RankData, Ratings ~ Hue + Saturation + Color value + Sharpness + Spectral Slope + Calories + Product weight + Price + (Hue||p) + (Saturation||p) + (Color value||p) + (Sharpness||p) + (Spectral Slope||p) + (Calories ||p) + (Product weight ||p) + (Price||p))*

c. Faces*: lmer (data=RankData, Ratings ~ Hue + Saturation + Color value + Sharpness + Spectral Slope + fWHR + Eyes distance + Nose eyes distance + (Hue||p) + (Saturation||p) + (Color value||p) + (Sharpness||p) + (Spectral Slope||p) + (fWHR ||p) + (Eyes distance||p) + (Nose eyes distance||p))*

S1 Table. Summary of Mixed-Effects Regression Models for Features Predicting Preference Ratings. Effects presented for lab (a), online (b) and replication (c) samples. Each column refers to a different category (fractals, snacks and faces), and each row refers to a different feature (Visual, Market and Facial features). For each model we calculated delta AIC score, as the AIC of the model minus the AIC of a null model (that is, a regression model for the dependent variables without any fixed or random factors). Brackets represent confidence intervals of $\alpha=0.05$. * $p<0.05$; ** $p<0.01$; *** $p<0.001$.

a

	Dependent variable: Ratings Lab	
	1	2
Hue	0.146***	−0.030***
	(0.127, 0.165)	(−0.046, −0.014)
Saturation	0.012	−0.051***
	(−0.012, 0.036)	(−0.068, −0.034)
Color Value	−0.126***	−0.013
	(−0.154, −0.097)	(−0.031, 0.004)
Sharpness	0.128***	0.078***
	(0.102, 0.153)	(0.062, 0.094)
Spectral Slope	0.122***	−0.037***
	(0.096, 0.147)	(−0.053, −0.021)
Calories		0.259***
		(0.238, 0.280)
Product Weight		0.096***
		(0.076, 0.116)
Price		0.097***
		(0.076, 0.118)
Constant	−0.009	0.023***
	(−0.025, 0.006)	(0.010, 0.037)
**Random Effect**		
n	235	307
Residual	0.909	0.891
Hue	0.064	0.000
Saturation	0.099	0.068
Color Value	0.150	0.089
Sharpness	0.126	0.000
Spectral Slope	0.123	0.000
Calories		0.131
Product Weight		0.077
Price		0.101
Observations	13,376	17,016
Log Likelihood	−18,046.710	−22,555.850
Akaike Inf. Crit.	36,127.420	45,163.700
Δ AIC	-2225.766	-4441.8269
Bayesian Inf. Crit.	36,254.940	45,364.990
Note:		*p<0.05; **p<0.01; ***p<0.001

b

	Dependent variable: Ratings Online		
	3	4	5
Hue	0.149***	−0.095***	0.031*
	(0.123, 0.176)	(−0.122, −0.069)	(0.005, 0.057)
Saturation	0.019	0.036*	0.082***
	(−0.022, 0.060)	(0.001, 0.072)	(0.057, 0.107)
Color Value	−0.154***	−0.048**	−0.110***
	(−0.204, −0.103)	(−0.083, −0.014)	(−0.141, −0.078)
Sharpness	0.102***	0.101***	0.006
	(0.064, 0.140)	(0.073, 0.129)	(−0.021, 0.033)
Spectral Slope	0.088***	−0.007	−0.078***
	(0.049, 0.128)	(−0.033, 0.020)	(−0.108, −0.047)
Calories		0.294***	
		(0.254, 0.334)	
Product Weight		0.026	
		(−0.009, 0.062)	
Price		0.007	
		(−0.026, 0.040)	
fWHR			−0.105***
			(−0.141, −0.069)
Eyes Distance			0.100***
			(0.070, 0.130)
Nose-eyes Distance			−0.097***
			(−0.127, −0.067)
Constant	−0.004	0.027*	0.021
	(−0.027, 0.018)	(0.005, 0.049)	(−0.001, 0.044)
**Random Effect**			
n	107	108	119
Residual	0.894	0.881	0.950
Hue	0.052	0.000	0.058
Saturation	0.148	0.129	0.046
Color Value	0.211	0.129	0.097
Sharpness	0.132	0.036	0.032
Spectral Slope	0.137	0.000	0.073
Calories		0.163	
Product Weight		0.096	
Price		0.074	
fWHR			0.081
Eyes Distance			0.000
Nose-eyes Distance			0.027
Observations	6,080	6,029	6,869
Log Likelihood	−8,160.558	−7,998.940	−9,507.729
Akaike Inf. Crit.	16,355.120	16,049.880	19,067.460
Δ AIC	NA	NA	NA
Bayesian Inf. Crit.	16,469.230	16,224.190	19,245.160

Note: *p<0.05; **p<0.01; ***p<0.001

c

	Dependent variable: Ratings Replication		
	6	7	8
Hue	0.104***	−0.059***	0.015
	(0.076, 0.131)	(−0.086, −0.033)	(−0.010, 0.039)
Saturation	0.012	0.016	0.073***
	(−0.027, 0.051)	(−0.018, 0.050)	(0.045, 0.101)
Color Value	−0.088***	−0.016	−0.066***
	(−0.136, −0.041)	(−0.049, 0.018)	(−0.098, −0.035)
Sharpness	0.056**	0.101***	0.001
	(0.015, 0.097)	(0.074, 0.128)	(−0.026, 0.027)
Spectral Slope	0.055*	−0.023	−0.073***
	(0.010, 0.100)	(−0.049, 0.004)	(−0.105, −0.042)
Calories		0.290***	
		(0.253, 0.327)	
Product Weight		−0.046**	
		(−0.079, −0.013)	
Price		0.038*	
		(0.003, 0.072)	
fWHR			−0.098***
			(−0.136, −0.060)
Eyes Distance			0.104***
			(0.073, 0.134)
Nose-eyes Distance			−0.075***
			(−0.105, −0.045)
Constant	0.003	0.034**	0.015
	(−0.019, 0.024)	(0.012, 0.057)	(−0.008, 0.038)
**Random Effect**			
n	114	109	115
Residual	0.896	0.897	0.953
Hue	0.071	0.000	0.000
Saturation	0.140	0.120	0.078
Color Value	0.199	0.123	0.087
Sharpness	0.164	0.000	0.000
Spectral Slope	0.186	0.000	0.076
Calories		0.142	
Product Weight		0.059	
Price		0.086	
fWHR			0.098
Eyes Distance			0.000
Nose-eyes Distance			0.000
Observations	6,463	6,086	6,640
Log Likelihood	−8,717.065	−8,150.678	−9,213.468
Akaike Inf. Crit.	17,468.130	16,353.360	18,478.940
Δ AIC	NA	NA	NA
Bayesian Inf. Crit.	17,583.290	16,527.910	18,655.760
Note:			*p<0.05; **p<0.01; ***p<0.001

S2 Model. Equations and results of model 2 logistic regressions. We fitted logistic mixed-effects regression models to explain binary choices. All regressors (i.e. Visual for fractals, Visual and Market for snacks and Visual and Facial for faces), served as fixed factors, with a random slope for each participant. All models were calculated in R (version 3.3.2), with the lme4 package (version 1.1-14). The R code of model 2 equations is presented for (a) fractals, (b) snacks and (c) faces. p=participants. Δ=difference of left item minus right item

a. Fractals: *glmer (data=RankData, family = binomial, Choices ~ (Δ rating│p) + Δ Ratings + Δ Hue + Δ Saturation + Δ Color value + Δ Sharpness + Δ Spectral Slope))*
b. Snacks: *glmer (data=RankData, family = binomial, Choices ~ (Δ rating│p) + Δ Ratings + Δ Hue + Δ Saturation + Δ Color value + Δ Sharpness + Δ Spectral Slope + Δ Calories + Δ Product weight + Δ Price))*
c. Faces: *glmer (data=RankData, family = binomial, Choices ~ (Δ rating│p) + Δ Ratings + Δ Hue + Δ Saturation + Δ Color value + Δ Sharpness + Δ Spectral Slope + Δ fWHR + Δ Eyes distance + Δ Nose eyes distance))*

S2 Table. Summary of Mixed-Effects Logistic Regression Models for Features Predicting Choice. Effects presented for online (a) and replication (b) samples. Each column refers to a different category (fractals, snacks and faces), and each row refers to a different feature (Visual, Market and Facial features). For each model we calculated delta AIC score, as the AIC of the model minus the AIC of a null model (that is, a regression model for the dependent variables without any fixed or random factors). Brackets represent confidence intervals of $\alpha=0.05$. * $p<0.05$; ** $p<0.01$; *** $p<0.001$.

a

	Dependent variable: Choices Online		
	3	4	5
Δ Ratings	0.926***	1.400***	0.819***
	(0.809, 1.042)	(1.250, 1.551)	(0.731, 0.908)
Δ Hue	0.062***	0.012	−0.014
	(0.038, 0.086)	(−0.017, 0.041)	(−0.035, 0.007)
Δ Saturation	0.020	−0.015	0.130***
	(−0.008, 0.049)	(−0.042, 0.013)	(0.109, 0.152)
Δ Color Value	0.006	−0.085***	−0.037**
	(−0.024, 0.036)	(−0.111, −0.059)	(−0.060, −0.013)
Δ Sharpness	0.046***	0.016	−0.051***
	(0.019, 0.073)	(−0.012, 0.044)	(−0.074, −0.028)
Δ Spectral Slope	0.005	−0.057***	−0.133***
	(−0.023, 0.034)	(−0.086, −0.029)	(−0.158, −0.108)
Δ Calories		0.133***	
		(0.103, 0.162)	
Δ Product Weight		0.005	
		(−0.028, 0.037)	
Δ Price		0.046**	
		(0.014, 0.079)	
Δ fWHR			−0.043**
			(−0.071, −0.016)
Δ Eyes Distance			0.094***
			(0.067, 0.121)
Δ Nose-eyes Distance			−0.069***
			(−0.095, −0.043)
Constant	−0.029	−0.188***	−0.028
	(−0.092, 0.034)	(−0.281, −0.096)	(−0.094, 0.039)
**Random Effect**			
n	107	108	119
Intercept	0.287	0.451	0.333
Δ Ratings	0.591	0.760	0.469
Observations	22,425	21,843	25,597
Log Likelihood	−12,286.090	−10,064.550	−14,291.710
Akaike Inf. Crit.	24,590.180	20,153.100	28,607.420
Δ AIC	-6497.7056	-10060.0949	-6877.4402
Bayesian Inf. Crit.	24,662.340	20,248.990	28,705.220
Note:			*p<0.05; **p<0.01; ***p<0.001

b

	Dependent variable: Choices Replication		
	6	7	8
Δ Ratings	1.004***	1.510***	0.841***
	(0.897, 1.110)	(1.360, 1.660)	(0.754, 0.927)
Δ Hue	0.079***	0.014	−0.002
	(0.056, 0.103)	(−0.016, 0.043)	(−0.023, 0.019)
Δ Saturation	0.035*	0.017	0.106***
	(0.006, 0.063)	(−0.010, 0.044)	(0.084, 0.127)
Δ Color Value	−0.024	−0.065***	0.004
	(−0.054, 0.005)	(−0.091, −0.038)	(−0.020, 0.027)
Δ Sharpness	0.039**	0.039**	−0.063***
	(0.012, 0.066)	(0.010, 0.067)	(−0.086, −0.039)
Δ Spectral Slope	−0.027	−0.040**	−0.136***
	(−0.055, 0.001)	(−0.069, −0.011)	(−0.161, −0.111)
Δ Calories		0.162***	
		(0.132, 0.192)	
Δ Product Weight		−0.065***	
		(−0.097, −0.032)	
Δ Price		0.041*	
		(0.008, 0.074)	
Δ fWHR			−0.032*
			(−0.060, −0.004)
Δ Eyes Distance			0.071***
			(0.043, 0.098)
Δ Nose-eyes Distance			−0.059***
			(−0.086, −0.032)
Constant	−0.008	−0.112***	0.035
	(−0.065, 0.050)	(−0.170, −0.053)	(−0.023, 0.092)
**Random Effect**			
n	114	109	115
Intercept	0.263	0.249	0.272
Δ Ratings	0.554	0.760	0.448
Observations	23,754	22,057	24,845
Log Likelihood	−12,771.080	−9,851.750	−13,891.020
Akaike Inf. Crit.	25,560.170	19,727.500	27,806.050
Δ AIC	-7371.3221	-10821.2792	-6634.788
Bayesian Inf. Crit.	25,632.850	19,823.520	27,903.490

Note: *p<0.05; **p<0.01; ***p<0.001

Bates, D., Mächler, M., Bolker, B., Walker, S. 2014. “Fitting Linear Mixed-Effects Models Using Lme4,” no. 1. https://doi.org/10.18637/jss.v067.i01.

Colley, W. N. 2002. “Colley’s Bias Free College Football Ranking Method: The Colley Matrix Explained.” *Working Paper*, 1–23. http://scholar.google.com/scholar?hl=en&btnG=Search&q=intitle:Colley+?+s+Bias+Free+College+Football+Ranking+Method+:+The+Colley+Matrix+Explained#0%5Cnhttp://scholar.google.com/scholar?hl=en&btnG=Search&q=intitle:Colley’s+bias+free+college+football+ranking.

Salomon, T., Botvinik-Nezer, R., Gutentag, T., Gera, R., Iwanir, R., Tamir, M., et al. 2018. “The Cue-Approach Task as a General Mechanism for Long-Term Non-Reinforced Behavioral Change.” *Scientific Reports*. https://doi.org/10.1038/s41598-018-21774-3.

Vieira, T.F., Bottino, A., Laurentini, A., De Simone, M. 2013. “Detecting Siblings in Image Pairs.” *The Visual Computer*.
